# Janus Quasi-Solid Electrolyte Membranes with Asymmetric Porous Structure for High-Performance Lithium-Metal Batteries

**DOI:** 10.1007/s40820-024-01325-4

**Published:** 2024-02-14

**Authors:** Zerui Chen, Wei Zhao, Qian Liu, Yifei Xu, Qinghe Wang, Jinmin Lin, Hao Bin Wu

**Affiliations:** Institute for Composites Science Innovation (InCSI) and State Key Laboratory of Silicon and Advanced Semiconductor Materials, School of Materials Science and Engineering, Zhejiang University, Hangzhou, 310027 People’s Republic of China

**Keywords:** Metal–organic frameworks, Mesoporous silicas, Quasi-solid electrolytes, Janus structure, Lithium-metal battery

## Abstract

Quasi-solid electrolytes (QSEs) based on nanoporous materials are promising candidates to construct high-performance Li-metal batteries (LMBs). However, simultaneously boosting the ionic conductivity (*σ*) and lithium-ion transference number (*t*_+_) of liquid electrolyte confined in porous matrix remains challenging. Herein, we report a novel Janus MOFLi/MSLi QSEs with asymmetric porous structure to inherit the benefits of both mesoporous and microporous hosts. This Janus QSE composed of mesoporous silica and microporous MOF exhibits a neat Li^+^ conductivity of 1.5 × 10^–4^ S cm^−1^ with *t*_+_ of 0.71. A partially de-solvated structure and preference distribution of Li^+^ near the Lewis base O atoms were depicted by MD simulations. Meanwhile, the nanoporous structure enabled efficient ion flux regulation, promoting the homogenous deposition of Li^+^. When incorporated in Li||Cu cells, the MOFLi/MSLi QSEs demonstrated a high Coulombic efficiency of 98.1%, surpassing that of liquid electrolytes (96.3%). Additionally, NCM 622||Li batteries equipped with MOFLi/MSLi QSEs exhibited promising rate performance and could operate stably for over 200 cycles at 1 C. These results highlight the potential of Janus MOFLi/MSLi QSEs as promising candidates for next-generation LMBs.

**Supplementary Information:**

The online version contains supplementary material available at 10.1007/s40820-024-01325-4.

## Highlights

- Janus quasi-solid electrolyte membranes with asymmetric porous structure were constructed, showing a high σ_Li+_ of 1.5 × 10^-4^ S cm^-1^ and a high* t*_+_ of 0.71.
- The solvation structures and ion transport dynamics in nanopores have been deciphered, manifesting a concentrated electrolyte-like structure and regulated transport behaviors.
- Quasi-solid NCM 622||Li cells have been demonstrated to stably cycle for 200 cycles at 1 C, and pouch cell has shown high tolerance for abuse.

**Supplementary Information:**

The online version contains supplementary material available at 10.1007/s40820-024-01325-4.

## Graphical abstract

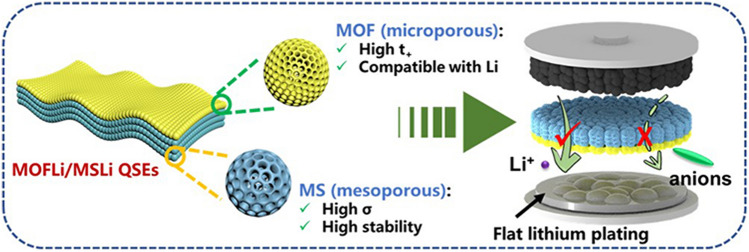

## Introduction

Nanoporous materials, exemplified by metal–organic frameworks (MOFs) [1, 2], covalent-organic frameworks (COFs) [3–5], and zeolites [6], have unique structural properties, including high surface area, tunable pore size, and adjustable chemical properties. These features enable them to construct quasi-solid electrolytes (QSEs) for next-generation rechargeable batteries such as Li-metal batteries (LMBs) [7–13]. By confining liquid electrolytes in microporous or mesoporous channels, QSEs derived from nanoporous materials exhibit decent room-temperature ionic conductivity (*σ*) (> 10^–4^ S cm^−1^) [14]. Moreover, the porous hosts have been found to inhibit the migration of anions [15, 16] and tailor the solvation structure of Li^+^ ions [17–19], resulting in high Li-ion transference number (*t*_+_) (typically exceeding 0.6) and enhanced stability. These features warrant QSEs as promising electrolyte candidates toward safe and durable LMBs.

Critical determinants of the cycling performance of Li-metal anode (LMA), as governed by Sand’s law [20], are the ionic conductivity and transference number of Li^+^ ions in electrolytes. Previous works have investigated the effects of pore structure [21], pore size [8, 22], and charge of the inner surface [8, 23] on ionic conductivity, concluding that larger pore size generally facilitates the migration of Li^+^. However, it is difficult to achieve both high *σ* and *t*_+_ when confining liquid electrolytes in a single type of porous material, as larger pore size weakens the confinement of liquid components and the ability to inhibit anion migration, compromising the advantages of QSEs. Additionally, though previous investigations have indicated the tailored solvation structure of Li^+^ ions in the nanopores [19], a clear depiction of the electrolyte structure at the atom level is still absent [24], impeding a comprehensive understanding of Li^+^ migration behavior.

**Scheme 1:**
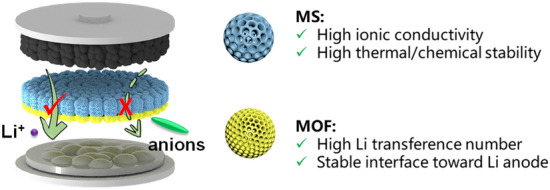
Schematic illustration of the Janus MSLi/MOFLi QSEs with an asymmetric porous structure

To achieve balanced *σ* and *t*_+_ in QSEs, we proposed novel Janus QSE membranes with asymmetric porous structure (denoted as MOFLi/MSLi QSEs) (Scheme 1). The primary layer was constructed using mesoporous silica (MS) nanoparticles, which allow the uptake of large quantity of liquid electrolyte and fast ionic transport in the mesopores [25]. Meanwhile, the MS is light weighted and chemically stable [26], which are helpful to improve the energy density and safety of LMBs. The second layer with a much thinner thickness was composed of metal–organic framework (MOF) nanoparticles with a microporous structure to regulate the solvation structure of electrolyte. UiO-66, a specific Zr-based MOF, was selected for its easy synthesis [27], ability to improve *t*_+_ [28], and excellent interfacial compatibility of the derived QSE for LMA [29]. Such asymmetric porous structure design overcomes the trade-off between *σ* and *t*_+_, and the resulting MOFLi/MSLi QSE displays a neat Li^+^ ion conductivity (*σ*_Li+_) up to 1.5 × 10^–4^ S cm^−1^ and high *t*_+_ of 0.71. The solvation structure of Li^+^ in MOF and MS was investigated by molecular dynamic (MD) simulation, depicting a desolvation structure and the preferred distribution of Li^+^ near the Lewis base O atoms of the porous hosts. Additionally, the nanoporous channels of MOFLi/MSLi QSEs effectively regulated ion flux and inhibited the growth of Li dendrites. Li||Li cells with MOFLi/MSLi QSEs demonstrated an extended cycling life, suggesting improved interfacial stability with LMA. NCM 622||Li batteries equipped with MOFLi/MSLi QSEs showed excellent rate performance and can stably work for 200 cycles at 1C. We further demonstrated a pouch cells (5.15 cm × 4.2 cm) with a total cathode capacity of 32.7 mA h, which could operate safety even under blending and cutting.

## Experimental Section

### Synthesis of MOF and MS Particles

#### Synthesis of UiO-66 nanoparticles

UiO-66 nanoparticles were synthesized according to our previous work [29]. Briefly, ZrCl_4_ (0.28 g, 1.372 mmol), H_2_BDC (0.20 g, 1.372 mmol), and 10 equivalents of benzoic acid (HBC) were dissolved in 70 mL DMF in a Teflon liner by stirring for about 30 min. The Teflon liner was then sealed in an autoclave and heated in an oven at 120 °C for 24 h. After cooling to room temperature, the solids were isolated by centrifugation, followed by being washed with DMF and ethanol several times, and then dried at 70 °C. The solids were further treated with 1 M HCl solution for 30 min to remove the coordinated HBC. The acid-treated solids were washed with deionized water, DMF, and ethanol and then dried under vacuum at 250 °C for 4 h to obtain activated UiO-66 nanoparticles.

#### Synthesis of MS nanoparticles

Mesoporous silica (MS) nanoparticles were synthesized following the method reported by Wang et al. [30]. Briefly, deionized water (20 mL) and ethanol (5 mL) were mixed to get a homogeneous solution. Then, 50 mg of triethanolamine and 0.2 g of cetyltriethylammnonium bromide were added into the above solution and stirred at 60 °C for 30 min. Subsequently, 2 mL of tetraethyl orthosilicate was added and stirred at 60 °C for another 2 h. 20 mL of ethanol was added into the white solution and cooled down to ambient temperature. The precipitate was collected by centrifugation and then washed with deionized water and ethanol several times. After being dried at 70 °C overnight, the powders were calcined at 550 °C for 5 h to get MS.

### Preparation of Quasi-solid Electrolytes

#### Preparing of MOFLi or MSLi QSE Pellets

MOF or MS (100 mg) and PVDF-HFP (100 mg) were mixed in dimethoxyethane (DME) to form a homogeneous solution and then dried at 50 °C under a vacuum. The solid product was pressed into a pellet with a diameter of 10 mm under a pressure of 10 MPa. The pellet was immersed in 1 M LiTFSI in polycarbonate (PC) solution for 4 h, and the QSE pellets were obtained.

#### Preparation of MOFLi or MSLi QSE membranes

MOF or MS (400 mg), glass fiber (20 mg), PVDF (100 mg), and LiTFSI (57 mg) were mixed in NMP (2.5 mL) to form a homogeneous gel. The gel was cast onto a cleaned glass plate. The casting gap of 100 μm was used for casting the membrane. Then, the gel was dried at 80 °C under a vacuum overnight to produce a free-standing membrane. The membrane was pouched into disks with a diameter of 18 mm and further dried at 120 °C for 10 h. Liquid electrolyte was then added based on the mass ratio (MOF or MS: LEs = 5: 4) obtained from QSE pellets.

#### Preparation of Janus MOFLi/MSLi QSE membrane

MS membrane was first prepared on a cleaned glass plate following the above procedure. To introduce the MOF layer, a homogeneous gel containing 100 mg of MOF, 25 mg of PVDF-HFP, and 1.25 mL of DME was prepared. The gel was then casted on the above MS membrane with a casting gap of 75 μm. After simply drying at ambient temperature, a free-standing membrane was harvested. The membrane was pouched into disks with a diameter of 18 mm and further dfried at 120 °C for 10 h. Liquid electrolyte was then added based on the mass ratio MOF/MS: LEs = 6: 4.

## Result and Discussion

### Design Principle and Structural Characterizations

**Fig. 1:**
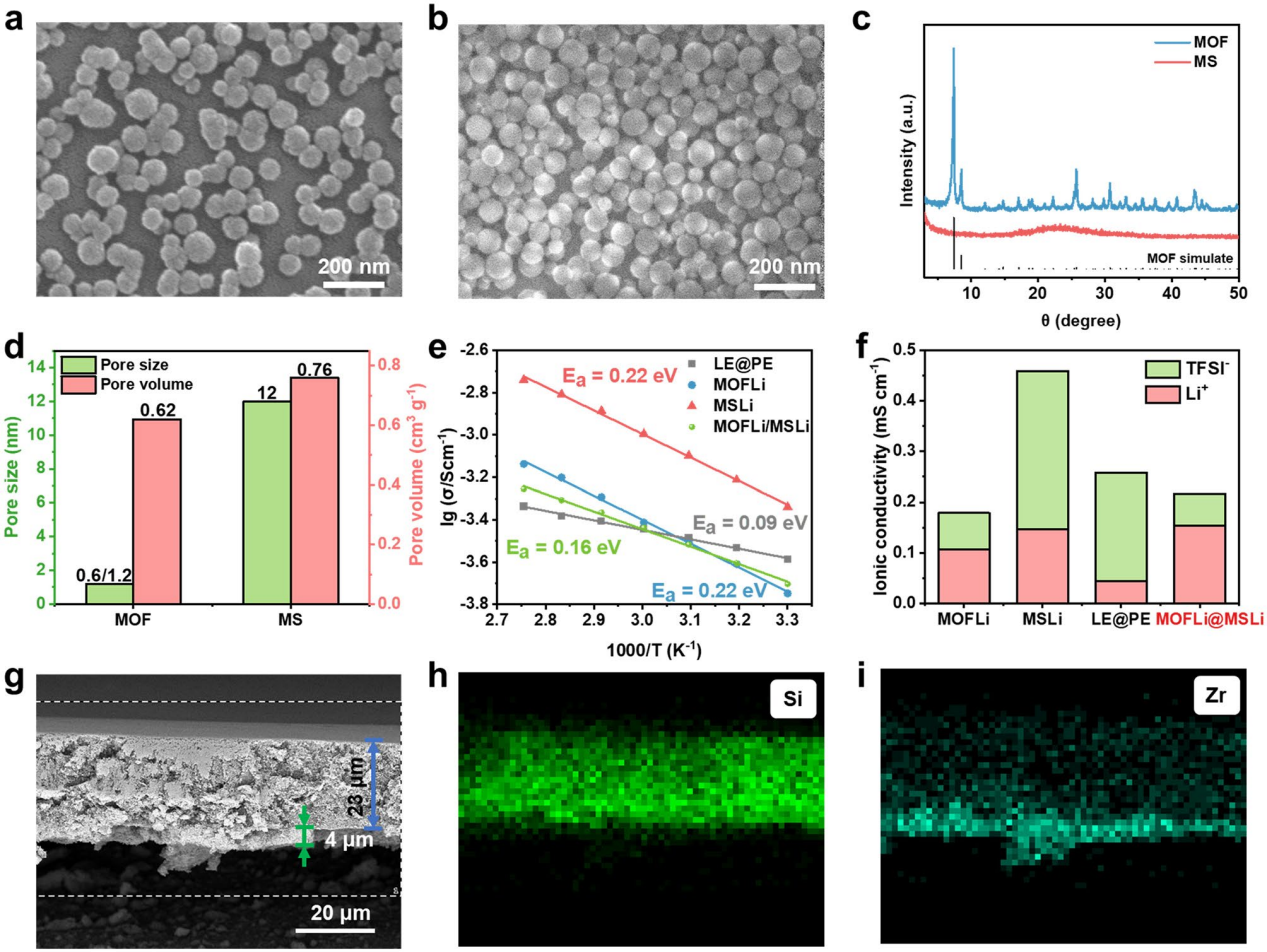
Structure characterization and electrochemical properties of MOF and MS particles. SEM image of **a** MOF particles and **b** MS particles. **c** PXRD patterns of the as-synthesized MOF and MS particles. **d** Pore size and pore volume of MOF and MS. **e** Corresponding Arrhenius plots of the ionic conductivity of LE in PE membrane (LE@PE, MOFLi QSEs, MSLi QSEs and MOFLi/MSLi QSEs. **f** Ionic conductivity corresponding to Li^+^ and TFSI^−^ of different samples based on EIS plots and *t*_+_ measurement. **g**-**i** SEM image and corresponding energy dispersive spectrometry (EDS) mapping of Janus MOF/MS membrane

MOF and MS particles were synthesized following previously reported methods [29, 30] and characterized by scanning electron microscopy (SEM) and powder X-ray diffraction (PXRD). SEM images demonstrated that both MOF and MS particles displayed uniform spherical morphology with diameters of about 80 nm (Fig. 1a, b). PXRD patterns indicated that the MOF particles possessed a crystal structure of UiO-66 [31] while MS particles were amorphous (Fig. 1c). Both the MOF and MS particles exhibited remarkable thermal stability as illustrated in Fig. S1. The observed mass loss below 300 °C can be attributed to the release of absorbed moisture [32–34]. Notably, the MOF particles start to decompose at approximately 500 °C, while the MS particles exhibited excellent thermal stability up to 700 °C. The pore structures of MOF and MS particles were then explored by nitrogen adsorption–desorption measurements (Figs. 1d, S2 and Table S1) [35]. The MOF particles displayed two kinds of pore configurations [36] with pore diameters of 0.6 and 1.2 nm, respectively, showing a pore volume of 0.62 cm^3^ g^−1^. The MS particles were mesoporous with a pore diameter of 12 nm and a pore volume of 0.76 cm^3^ g^−1^. The MS with low tap density (Fig. S3) minimizes the extra weight brought by electrolytes, which is advantageous when comparing with inorganic solid electrolytes of high density.

In order to obtain QSEs, MOF and MS powers were firstly compressed into pellets with 20 wt% PVDF-HFP (functioned as binder). The obtained MOF@PVDF-HFP and MS@PVDF-HFP displayed a slight decrease in pore size and pore volume (Fig. S2 and Table S1), but still exhibited microporous and mesoporous structures, respectively. The pellets were subsequently soaked in 1 M LiTFSI in PC solution. After fully adsorbed with liquid electrolytes, QSE pellets were harvested (denoted as MOFLi QSE and MSLi QSE). Benefiting from the larger pore size and pore volume, MSLi QSEs were able to adsorb more liquid electrolyte (39 wt%) than MOFLi QSEs (23 wt%) (Fig. S4). The ionic conductivities of MOFLi QSEs and MSLi QSEs were investigated using electrochemical impedance spectroscopy (EIS) (Figs. 1e and S5). MOFLi QSE and MSLi QSE displayed room-temperature ionic conductivity (*σ*_total_) of about 1.6 × 10^–4^ and 4.6 × 10^–4^ S cm^−1^, respectively. Additionally, PVDF-HFP-based gel electrolyte exhibited room-temperature ionic conductivity of only 1.6 × 10^–5^ S cm^−1^ (Fig. S6), which contributed little to the ionic conductivity of QSE. Though the pure liquid electrolyte (LE) (1 M LiTFSI in PC) demonstrated *σ*_total_ up to 5.6 × 10^–3^ S cm^−1^, liquid electrolyte in commercial PE membrane only delivered *σ*_total_ of 2.6 × 10^–4^ S cm^−1^. Both the MS and MOF surfaces of the QSE exhibit remarkable wettability with liquid electrolyte as confirmed by the contact angle measurement (Fig. S7) [37]. The contact angles of LE on MS and MOF membranes are notably smaller than that on PE membrane (18.7° and 23.5° vs. 71.6°). Porosity of the MOF and MS pellets estimated by the LE uptakes is 28% and 49%, respectively, while that of PE membrane is 35% (Table S2). The high LE uptake of MS membrane and its excellent wettability with LE explain the higher ionic conductivity of MSLi QSE compared with LE in PE membrane.

Lithium-ion transference number (*t*_+_) was further measured to assess the contributions of Li^+^ and TFSI^−^ ions to total ionic conductivity (Figs. 1f and S8). Benefiting from the microporous structure and strong MOF-LE interaction, MOFLi QSE is capable to restrict the migration of anions and delivered *t*_+_ up to 0.60, while MSLi QSE and liquid electrolyte in PE membrane could only offer *t*_+_ of 0.32 and 0.17, respectively. With both *σ*_total_ and *t*_+_ taken into consideration, MSLi QSE displays the highest neat Li^+^ conductivity (*σ*_Li+_) of 1.5 × 10^–4^ S cm^−1^ while MOFLi QSE demonstrates the lowest TFSI^−^ conductivity (*σ*_TFSI-_) of 7.2 × 10^–5^ S cm^−1^.

To develop QSEs with convenient Li^+^ migration and restrained TFSI^−^ transport, Janus QSEs with asymmetric porous structure were proposed by applying MS as the major layer to conduct Li^+^ and MOF as the functional layer to hinder TFSI^−^ migration. The Janus MOF/MS membrane was fabricated using a layer-by-layer tape casting method (see Experimental section and Fig. S9), which enables large-area fabrication of the Janus membrane (Fig. S10). The Janus membrane consists of a relatively thick MS layer to ensure high ionic conductivity and a relatively thin MOF layer to inhibit the migration of anions [10, 38]. As demonstrated in Fig. 1g-i, MOF/MS membrane finally delivered asymmetric double-layer structure with a total thickness of about 27 μm, of which the MS layer was 23 μm and the MOF layer was 4 μm. The MOF/MS membrane is dense, and the upper surface of the membrane (MOF side) exposed to air during drying was relatively rough (Fig. S11). High thermal stability of MOF and MS enabled the MOF/MS membrane to maintain the original structure up to 200 °C, while commercial PE membrane suffered from dramatic shrink (Fig. S12). Meanwhile, the Yang’s modulus of the MOFLi/MSLi membrane was examined using atomic force microscopy (AFM) as shown in Fig. S13. The average Yang’s moduli on MSLi and MOFLi sides are 1.66 and 1.43 GPa, respectively, which are notably higher than that of PE membrane (160 MPa). The Janus structure enabled MOFLi/MSLi QSE to deliver *σ*_total_ of 2.2 × 10^–4^ S cm^−1^ and *t*_+_ up to 0.71, translating to *σ*_Li+_ of 1.5 × 10^–4^ S cm^−1^, which was comparable with that of MSLi QSEs. The simultaneously displayed high Li^+^ ion conductivity and low TFSI^−^ ion conductivity suggested the efficient asymmetric structure design of MOFLi/MSLi QSEs.

### Solvation Structure and Ion Transport Dynamic

**Fig. 2:**
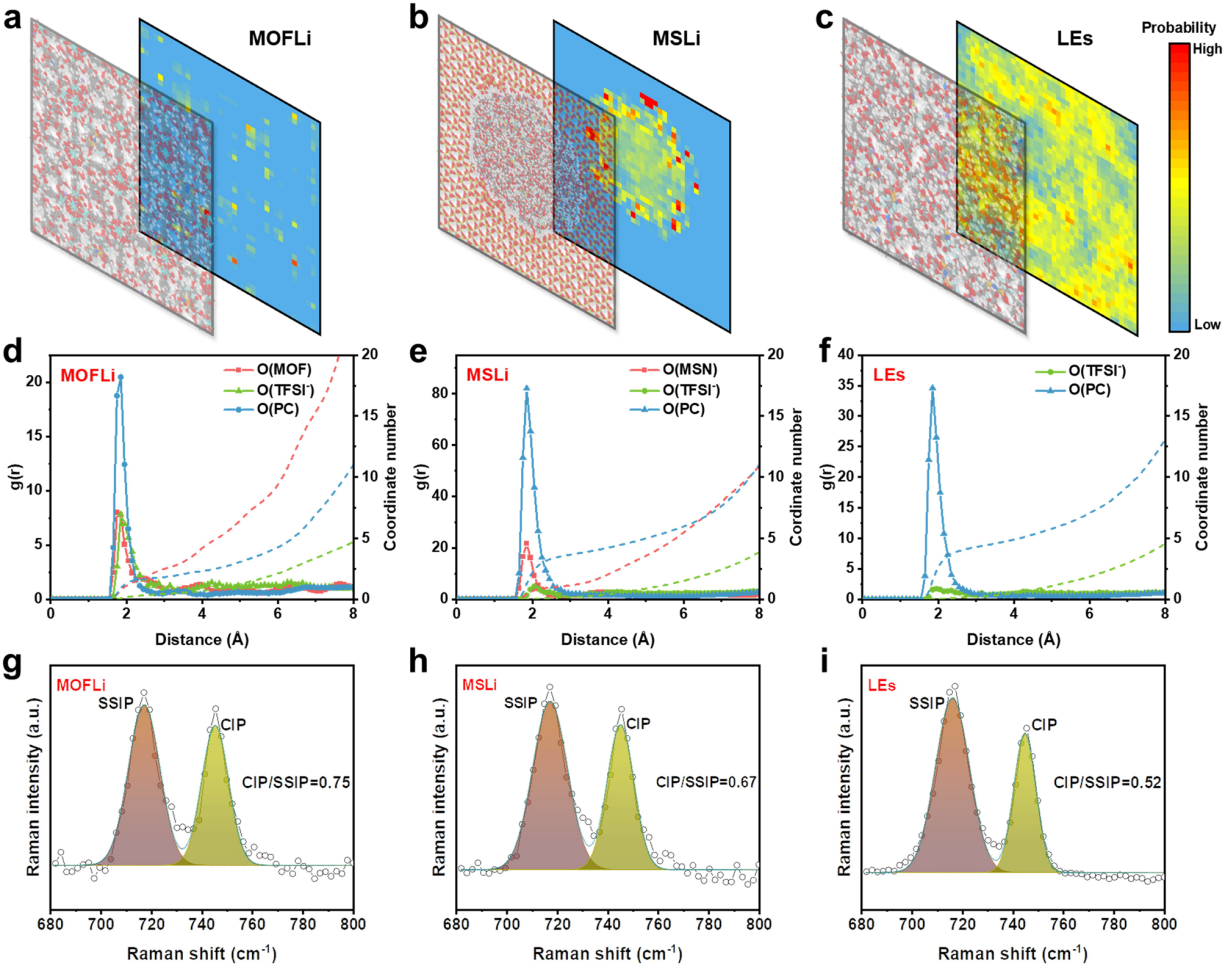
Molecular dynamic (MD simulations of different electrolytes. The final snap of the models and the distribution of Li^+^ during the whole simulation procedure of **a** MOFLi, **b** MSLi, and **c** LEs. Radial distribution function (RDF) and Raman spectra of **d**, **g** MOFLi, **e**, **h** MSLi and **f**, **i** LEs

The solvation structure and ion transport dynamics of QSEs were analyzed by molecular dynamic (MD) simulations [39]. Three models, namely MOFLi, MSLi, and LEs, were set up for MD simulations (Fig. 2a-c). The change in the solvation structure of Li^+^ ions in different electrolytes was revealed by radial distribution function (RDF) (Fig. 2d-f). In LE, Li^+^ was fully solvated by PC molecules, with an average coordination number (CN) of 4.4. When confined in porous hosts, due to the spatial restriction of pore size, a de-solvation process took place on Li^+^ ions to generate a smaller solvation sheath, reflected by the reduction of CN of O(PC) (3.8 and 1.9 for MSLi and MOFLi, respectively). Moreover, the Lewis base atoms of the porous hosts (O(Zr–O–C) from MOF and O(Si–O–Si) from MS) seem to participate in the solvation of Li^+^ (Fig. S14), delivering coordination number of 2.7 and 1.3, respectively. Such interaction between porous hosts and Li^+^ ions results in preferential location of Li^+^ ions near the O atoms in the pore wall [40–42], contrasting with their homogeneous distribution in LE (Figs. 2a-c and S15). Additionally, TFSI^−^ competed to enter the solvation sheath of Li^+^ due to the decrease in CN of PC, reflected by the increase in the ratio of g(r) attributed to O(TFSI^−^) and O(PC) (Fig. S16). The change in the solvation structure of Li^+^ leads to different migration mechanism [24], which is evidenced by the change in activation energy in Arrhenius plots (0.22, 0.22, and 0.09 eV for MOFLi QSE, MSLi QSE, and LE in PE membrane, respectively) (Fig. 1d).

Furthermore, to confirm the unique solvation structures of QSEs obtained by MD simulations, Raman spectra were performed on different electrolytes (Figs. 2g-i and S17). Peaks at around 700–780 cm^−1^ were attributed to the vibrations of TFSI^−^ anions, which are affected by the solvation with Li^+^ ions [7, 17, 43]. Peaks at 722 cm^−1^ were assigned to solvent-separate ion pairs (SSIP) while peaks at 746 cm^−1^ were attributed to the formation of contact ion pairs (CIP) with Li^+^ ions. Increasing the concentration of liquid electrolyte (from 0.3 to 3 M) leads to the enhanced intensity of CIP-peaks (Fig. S17), which reflects the change of solvation structure [7]. When liquid electrolyte was confined in the nanoporous host, the solvation sheath of Li^+^ would be tailored and more TFSI^−^ ions would coordinate with Li^+^ ions to form CIPs. The higher ratio between CIPs and SSIPs in MOFLi and MSLi than 1 M LiTFSI/PC electrolyte revealed that a concentrated-electrolyte-like solvation structure is created in the nanopore channels, which is helpful to achieve an anion-induced SEI on LMA [19, 44]. The higher proportion of CIPs in QSEs might partly explain the high *t*_+_ due to the restricted migration of anions as well as the lower Li^+^ conductivity compared with pure LE.

Mean squared displacement (MSD) reflects the diffusion ability of Li^+^ and TFSI^−^ in MOFLi QSE, MSLi QSE, and pure liquid electrolyte (LE) [40] (Fig. S18). The largest MSD value of Li^+^ and TFSI^−^ in LE indicated their highest mobility in pure liquid phase. When liquid electrolyte is confined in porous hosts, the diffusion of both Li^+^ and TFSI^−^ was restricted due to the restriction of the porous matrix and interaction with the Lewis base O atoms, yielding ionic diffusion coefficients (*D*_diff_) in the order of *D*_LE_ > *D*_MSLi_ > *D*_MOFLi_. Nonetheless, the diffusion of Li^+^ and TFSI^−^ was impeded by porous hosts to different extents. When confined in microporous MOF particles, the migration of TFSI^−^ ions was tremendously restricted since the size of anions (about 0.8 nm) is close to the pore window size of the MOF particles. The trend in how the ion transport dynamics change in the porous hosts predicted by MD simulations is consistent with the experimental results, confirming the effective modulation of ion transport by specific porous hosts.

### Ion Flux Regulation and Lithium Deposition

**Fig. 3:**
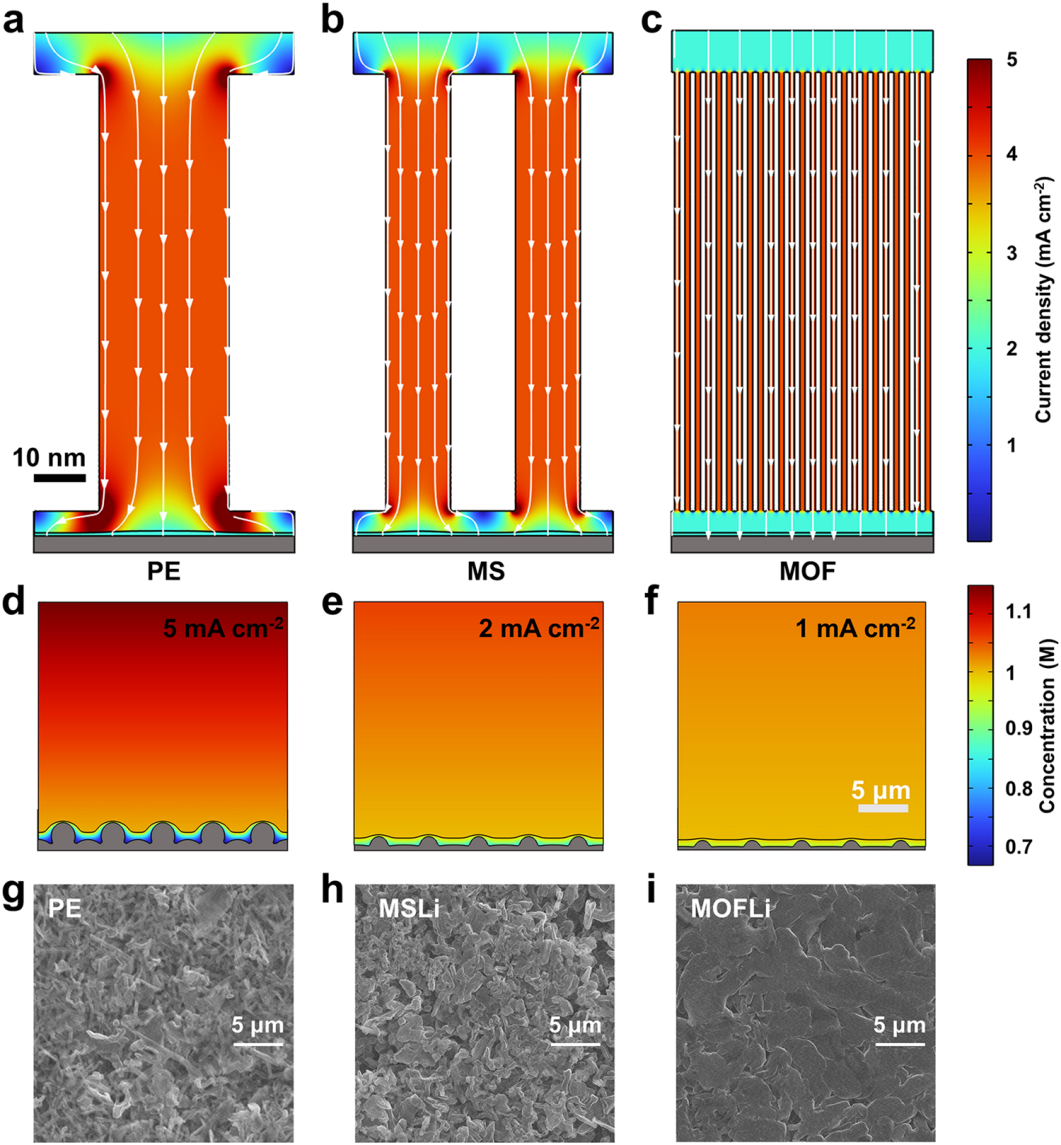
Finite element method (FEM) simulations of different types of electrolytes. Ion flux distribution when employing **a** LEs in PE membrane, **b** MSLi QSEs and **c** MOFLi QSEs. Simulation of the deposition of Li^+^ at a local current density of **d** 5 mA cm^−2^, **e** 2 mA cm^−2^, and **f** 1 mA cm^−2^. **g**-**i** The morphology of the deposited Li under a current density of 0.5 mA cm^−2^ and a capacity of 5 mAh cm.^−2^

Apart from the high *σ*_Li+_ and high *t*_+_ by integrating the merits of mesoporous MS and microporous MOF, MOFLi/MSLi QSEs also inherit their advantage of ion flux regulation, which is also crucial for achieving homogeneous Li deposition and preventing the growth of Li dendrites. To investigate the effect of the pore structure on ion flux regulation, the finite element method (FEM) was used to simulate ion flux in different electrolytes [29, 45, 46] (Figs. 3a-c and S19). In the PE membrane with a large pore size, obvious ion flux turbulence with extremely high current density in certain areas was observed, which is hazardous for Li deposition since the concentration polarization is exacerbated and dendrites grow quickly. In contrast, the nanoporous structures of MSLi and MOFLi QSEs efficiently homogenize the ion flux distribution. The influence of local current density on the growth of Li dendrites was further simulated (Figs. 3d-f and S20). With the increase in current density, obvious Li protrusions formed on top of the electrode, which would gradually evolve into dendritic structures. The morphologies of the deposited Li on Cu collectors using various electrolytes corroborated with the FEM simulation results (Fig. 3g-i). PE membrane with liquid electrolyte was not capable to regulate ion flux but accelerated the growth of Li dendrites. In contrast, homogenous nanoporous channels, particularly the microporous channels in MOFLi QSE, favor the uniform and dense Li deposition, which would improve the efficiency and durability of LMA. Similar homogeneity in Li deposition can be achieved by MOFLi/MSLi QSE once the MOFLi layer is in close contact with LMA as discussed shortly.

### Cycling Performance of LMBs

**Fig. 4:**
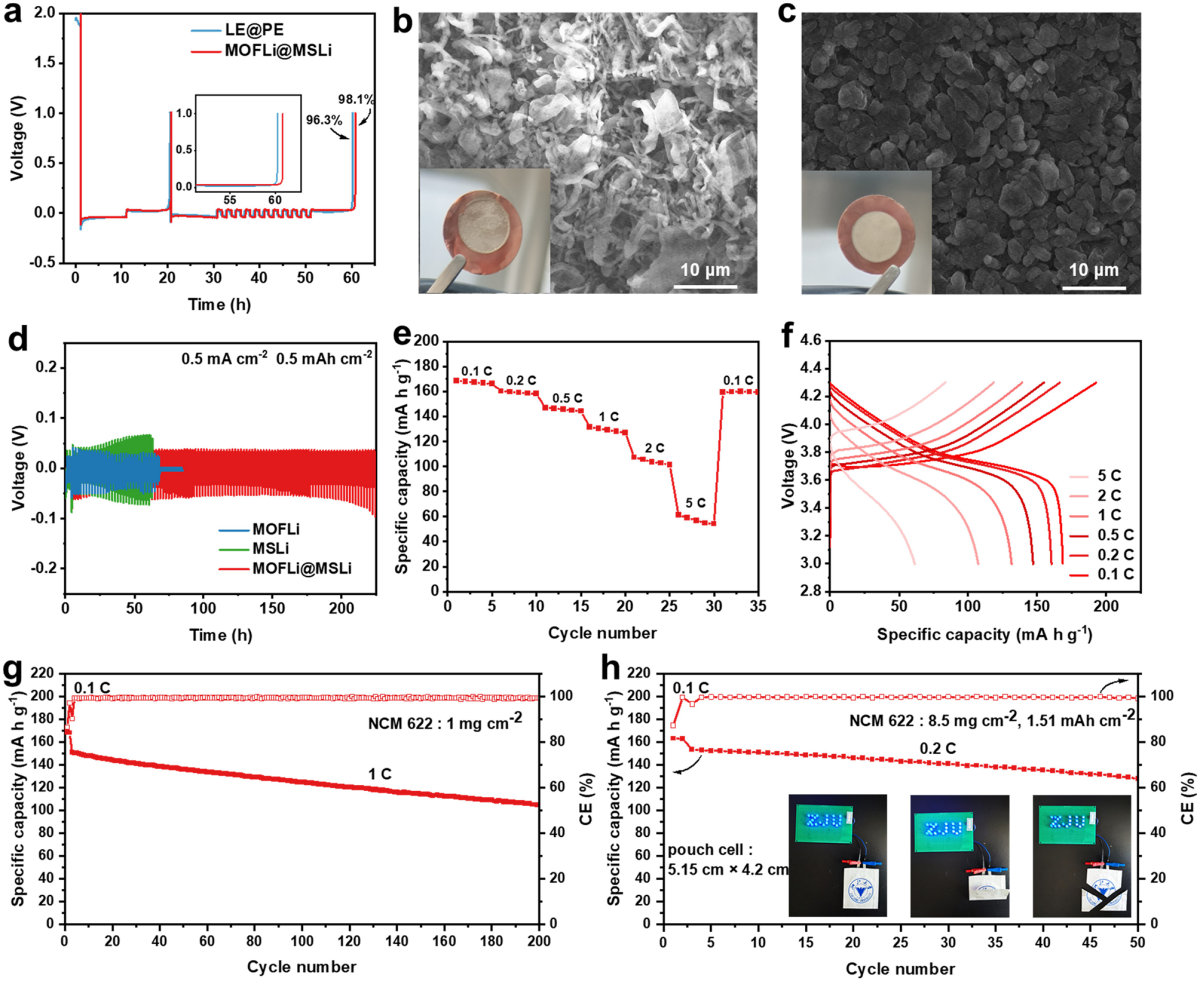
Half cells and full batteries performance. **a** Average CE of Li||Cu cells using PE membrane and MOFLi/MSLi QSEs at a current density of 0.5 mA cm^−2^. The morphology of the deposited Li with a depositing capacity of 2 mAh cm^−2^ on Cu when using **b** PE membrane and **c** MOFLi/MSLi QSEs. The inset images were the optical picture of the deposited Li. **d** Cycle performance of Li||thin Li symmetric cells at a current density of 0.5 mA cm^−2^. **e**–**f** Rate performance of NCM 622|MOFLi/MSLi|Li batteries and the corresponding voltage profile. **g** Cycling performance of NCM 622||Li batteries at 1 C. **h** Cycling performance of pouch cell with high mass loading cathode. The inset was the safety illustration of pouch cell with MOF/MS electrolyte under abuse use

Li||Li cells equipped with advantaged MOFLi/MSLi QSE membrane showed much extended cycling life than PE membrane with 1 M LiTFSI in PC liquid electrolyte, confirming the ability of MOFLi/MSLi QSE to improve the performance of LMA (Fig. S21). To construct the full cells of LMB, QSE made with commercial liquid electrolyte (1 M LiPF_6_ in EC/DMC (1:1 by volume) with 5% FEC) was further evaluated. Li||Cu cells were assembled to evaluate the Coulombic efficiency (CE) of Li deposition/stripping in different electrolytes (Fig. 4a). When equipped with MOFLi/MSLi QSE, a high average CE up to 98.1% was harvested, compared with the CE of 96.3% when using a PE membrane and liquid electrolyte. Furthermore, MOFLi/MSLi QSE yielded a lower nucleation potential of 72 mV compared with that of 120 mV in liquid electrolyte (Fig. S22), which might attribute to the affinity of the MOF layer toward Li^+^ [29]. As illustrated in Figs. 4b, c and S23, after electrochemically depositing 5 mAh cm^−2^ of Li on bare Cu current collector, macroscopically uneven Li deposition was observed when using PE membrane and liquid electrolyte, compared with the flat surface harvested from the cell equipped with MOFLi/MSLi QSE. SEM images illustrated serious Li dendrite growth when using PE membrane. The homogenous Li deposition with columnar shape enabled by MOFLi/MSLi QSE could be attributed to the high *σ*_Li+_ and *t*_+_, regulated ion flux, and reduced nucleation barrier.

Critical current density (CCD) of MOFLi/MSLi QSEs was assessed to evaluate the capability to operate at high current densities. As shown in Fig. S24, MOFLi and MSLi QSEs experienced a short circuit at current densities of 0.7 and 1.2 mA cm^−2^, respectively. In contrast, Janus MOFLi/MSLi QSEs demonstrated high tolerance to increasing current densities up to 2.1 mA cm^−2^. Li||thin Li (25 μm) cells were also assembled to evaluate the utilization of Li (Fig. 4d). Cells using MOFLi and MSLi QSEs suffered from quick failure, probably relating to the inefficient *σ*_Li+_ or *t*_+_ when operating under rigorous condition. In contrast, cell using MOFLi/MSLi QSE can stably work for 230 h with a low overpotential of 21 mV. X-ray photoelectron spectroscopy (XPS) was performed on the lithium anode after 10 cycles to investigate the composition of the SEI layer. As shown in Fig. S25, all samples exhibited C 1 *s* peaks at 286.4, 288.7, and 289.8 eV, corresponding to C–O, C=O, and Li_2_CO_3_, respectively, which originated from the decomposition of carbonate solvent. The F 1 *s* peaks at 684.8 and 686.6 eV were identified as signals of LiF and Li_*x*_PO_*x*_F_*z*_, respectively, resulting from the decomposition of lithium salt [47–49]. Remarkably, the content of Li_2_CO_3_ is lower on Li cycled with MOFLi/MSLi QSE compared to those with MOFLi and MSLi, which suggest less solvent decomposition. New peaks corresponding to C–F in the C 1*s* spectrum (291.2 eV) and F 1*s* spectrum (688.6 eV) might come from the decomposition of PVDF-HFP in the MOF layer.

The oxidation stability of MSLi and MOFLi QSEs was assessed through linear sweep voltammetry (LSV) measurements. As depicted in Fig. S26, MSLi QSE exhibited superior cathodic stability up to 4.6 V, surpassing the stability of MOFLi QSE (4.25 V). The exceptional oxidation stability of MSLi QSE enables the use of high-voltage cathode for high-energy LMBs. Herein, full cells of LMB using high-voltage cathode (NCM 622) were assembled. The full cell equipped with MOFLi/MSLi QSE showed favorable rate performance, delivering a specific capacity of 168, 160, 147, 131, and 107 mAh g^−1^ at 0.1, 0.2, 0.5, 1, 2, and 5 C, respectively (Fig. 4e, f). NCM 622||Li batteries (NCM 622 loading: 1.0 mg cm^−2^) could stably work for 200 cycles with 70% capacity retention (Fig. 4g), maintaining a CE of 99.5%. The Janus MOFLi/MSLi QSEs are also compatible with other high-energy cathodes such as LiCoO_2_ (LCO). As shown in Fig. S27, LCO||Li batteries demonstrated excellent rate performance, delivering specific capacities of 143, 136, 124, 110, 92, and 77 mAh g^−1^ at 0.1, 0.2, 0.5, 1, 2, and 3 C, respectively. LCO||Li batteries (LCO loading: 0.8 mg cm^−2^) also exhibited stable operation for 200 cycles with 83% capacity retention. Large-size pouch cell (5.15 cm × 4.2 cm) with high mass loading cathode (8.5 mg cm^−2^, 1.51 mAh cm^−1^) could also stable work at 0.2 C. Benefiting the solid-like nature of MOFLi/MSLi QSEs and confinement of liquid electrolyte in nanopores, pouch cell showed excellent safety and high tolerance toward abused uses such as bending and cutting (Fig. 4h).

## Conclusion

In this work, we present a Janus MOFLi/MSLi QSEs with asymmetric porous structure for LMBs. The Janus electrolyte was found to inherit the advantages of mesoporous MS and microporous MOF, maintaining both high *σ*_Li+_ of 1.5 × 10^–4^ S cm^−1^ and high *t*_+_ of 0.71. MD simulations revealed a concentrated-electrolyte-like solvation structure and preferred distribution of Li^+^ near the Lewis-base O atoms in the nanoporous channels, as well as regulated ion transport dynamics. Meanwhile, the nanoporous structure would homogenize the Li deposition by regulating the ion flux. As a result, Li||Cu cells equipped with the Janus MOFLi/MSLi QSEs delivered a high CE of 98.1%, compared with 96.3% when using PE membrane. NCM 622||Li quasi-solid batteries assembled with MOFLi/MSLi QSEs showed promising rate performance and stably worked for 200 cycles at 1 C. This work demonstrates how to regulate the solvation structure and ion transport by proper pore structure design for QSEs, which enables highly selective Li^+^ ion conduction with high conductivity.

## Supplementary Information

Below is the link to the electronic supplementary material.

Supplementary file1 (PDF 1751 KB)
