# Supplementary material for: Janus Quasi-Solid Electrolyte Membranes with Asymmetric Porous Structure for High-Performance Lithium-Metal Batteries
Source: Nanomicro Lett. 2024 Feb 14;16:114. doi: 10.1007/s40820-024-01325-4 (PMC10866846; doi:10.1007/s40820-024-01325-4)
Supplement: Supplementary file 1 — Supplementary file1 (PDF 1751 KB) [file 40820_2024_1325_MOESM1_ESM.pdf]

Supporting Information for

Janus Quasi-Solid Electrolyte Membranes with Asymmetric Porous Structure for High-Performance Lithium-Metal Batteries

Zerui Chen[1], Wei Zhao[1], Qian Liu[1], Yifei Xu[1], Qinghe Wang[1], Jinmin Lin[1], Hao Bin Wu[1, *]

[1]Institute for Composites Science Innovation (InCSI) and State Key Laboratory of Silicon and Advanced Semiconductor Materials, School of Materials Science and Engineering, Zhejiang University, Hangzhou 310027, P. R. China

*Corresponding author. E-mail: hbwu@zju.edu.cn (Hao Bin Wu)

S1 Experimental Procedures

S1.1 Materials

Zirconium tetrachloride ($ZrCl_4$), benzoic acid (HBC), tetraethyl orthosilicate (TEOS), N-methyl pyrrolidone (NMP) were purchased from Aladdin Industrial Corporation. 1,4-dicarboxybenzene (H_2BDC), N,N-Dimethylformamide (DMF), and triethanolamine ($C_6H_{15}NO_3$) were purchased from Shanghai Macklin Biochemical Technology Co., Ltd. Ethanol, hydrochloric acid (HCl), and hexadecyltrimethylammonium bromide (CATB) were purchased from China National Medicines Corporation Ltd. Lithium bis(trifluoromethanesulfonyl)imide (LiTFSI) were purchased from Guotai super power. All the chemicals were used directly without further purification.

S1.2 Preparation of Cathode

Commercial cathode material (NCM 622), super-P and PVDF were mixed in NMP with a mass ratio of 8:1:1 to obtain the cathode mixture. The cathode mixture was coated on carbon-coated aluminum foil. The prepared electrode was dried at 80 °C under a vacuum overnight. The electrode films were punched into disks with a diameter of 12 mm. The loading of the active material was around 1 mg cm^{-2}. The NCM 622 cathode electrodes with high active mass loading (8.8 mg cm^{-2}) were pursued from Guangdong Canrd New Energy Technology Co.,Ltd. The voltage window for the batteries equipped with NCM 622 cathodes was set to 3 - 4.3 V.

S1.3 Characterization

X-ray diffraction patterns were acquired by an X-ray diffractometer (EMPYREAN PANalytical) with Cu-Kα radiation (λ=1.54 Å). The morphologies of the samples were observed by field-emission scanning electron microscopy (SEM) (Phenom, PW-100-060). Nitrogen adsorption–desorption measurements at 77 K were performed on a TriStar II 3020 surface area analyzer. Before analysis, samples were degassed at 120 °C for 24 h. The pore sizes of MOF and MS were determined by density function theory (DFT) model and Barret-Joyner-Halenda (BJH) model, respectively. Raman spectra was obtained on a HORIBA LabRAM HR Evolution Raman spectrometer with a 473 nm laser. Thermal gravimetric analysis (TGA) profiles were obtained on a SDT Q600 V8.2 Build 100 with a heating rate of 10 °C min^{-1} in air. The atomic force microscopy (AFM, MFP-3D Origin,

Asylum Research, Oxford Instruments) was used to analyze Young's modulus of the samples. Uniformly distributed 100 points were sampled for Yang's Modulus testing. The surface composition and valence state were analyzed by X-ray photoelectron spectroscopy (XPS, Thermo SCIENTIFIC ESCALAB XQI) with Al Kα radiation. All XPS spectra were calibrated by shifting the detected adventitious carbon C 1s peak to 284.8 eV. Contact angle measurements were performed on JY-82C. The porosity was assessed by immersing the porous matrix in a liquid electrolyte solution (1M LiTFSI in PC) and calculating the volume ratio between the absorbed electrolytes and the porous matrix.

S1.4 Electrochemical Measurements

Ionic conductivity was tested with an electrochemical workstation (BioLogic). The ionic conductivity was determined using electrochemical impedance spectroscopy (EIS) with pellets or membranes between two stainless steel (SS) electrodes in 2032-type coin cells. The frequency range was from 100 kHz to 0.1 Hz and the AC amplitude was 10 mV. The ionic conductivity (σ, S cm^{-1}) was calculated by using the endpoint of the semicircle as the ion resistivity (R, Ohm), thickness (L, cm), and area (S, cm^2) of the pellets or membranes based on $\sigma = L/(R\times S)$. Symmetric cells were fabricated using two pieces of the Janus electrolyte to ensure MOF side was placed toward lithium metal electrode. Electrochemical window was measured by linear sweep voltammetry (LSV) using Li|electrolyte|SS cells with a scan rate of 1 mV s^{-1} range from open voltage to 5.0 V.

For the measurement of average CE, 5 mA h cm^{-2} Li was first plated on Cu foil and stripped to 1.0 V at a current density of 0.5 mA cm^{-2}. Then plate quantitative Li reservoir (Q_t = 5 mA h cm^{-2}) on Cu foil, repeatedly strip/plate Li with an area capacity of 0.5 mA h cm^{-2} (Q_c) for n cycles (n=10), and finally trip all the residual Li (Q_s) to a cutoff voltage of 1.0 V at a current density of 0.5 mA cm^{-2}. The average CE over the n cycle was calculated based on $CE_{ave} = (Q_s+nQ_c)/(Q_t+nQ_c)$.

S1.5 Simulation of Current Density and Lithium Dendrite

Li-ion flux and deposition were simulated by using the finite element solver of COMSOL Multiphysics. For the simulation of Li-ion flux when using different electrolytes, simplified 2D models were first built (Fig. S9), where the bottom of the model represented the Li anode. An SEI layer with a thickness of 1 nm was constructed on the surface of the anode. The geometry parameters were presented in Fig. S12. The diffusion coefficients of Li^+ in PE, MS, MOF and SEI layer were set as 5 × 10^{-7}, 1.5 × 10^{-6}, 1 × 10^{-6}, and 5 × 10^{-9} cm^2 s^{-1}, respectively. The diffusion coefficients of anions were set as 2 × 10^{-6}, 3 × 10^{-6}, 7 × 10^{-7}, and 2 × 10^{-8} cm^2 s^{-1}, respectively. The initial concentration of Li^+ was set to 1 M and the average current density was set as 2 mA cm^{-2}.

As for the simulation of the growth of lithium dendrite under different densities, simplified models with spherical cap-shaped nucleus were built, whose geometry parameters were illustrated in Fig. S13. The diffusion coefficient of Li^+ in electrolyte and SEI layer were set as 5 × 10^{-7} cm^2 s^{-1}, 5 × 10^{-9} cm^2 s^{-1}, respectively, while that of anions were 2 × 10^{-6} cm^2 s^{-1} and 2 × 10^{-8} cm^2 s^{-1}, respectively. The average current density was set as 0.5 mA cm^{-2}, 2 mA cm^{-2} and 5 mA cm^{-2}, respectively. Note that all models were ideal and cannot fully represent the real situation.

S1.6 Molecular Dynamic (MD) Simulations

MD simulations were performed based on the Large Scale Atomic/Molecular Massively Parallel

Simulator (LAMMPS) code [S3]. Three models of different types of electrolytes were built for simulation: 1) MOFLi QSEs: UiO-66 crystal with size of 42.0 Å × 42.0 Å × 42.0 Å was first built, followed by the adding of 32 LiTFSI and 386 PC based on the mass ratio of the experiment; 2) MSLi QSEs: SiO_2 crystal with size of 73.65 Å × 76.5 Å × 54.02 Å was built, following by deleting the atoms inside the cylinder with a diameter of 5.0 nm to build MS. Liquid electrolyte (1 M LiTFSI in PC) was then filled the channel of MS to get MSLi. 3) Liquid electrolytes: A box with a size of 33.9 Å × 33.9 Å × 33.9 Å was filled with 20 Li^+, 20 $TFSI^-$, and 240 PC to simulate 1 M LiTFSI/PC solution.

The OPLS-AA force field was used to describe the energy potential of Li^+ [S4, S5] and UFF force field was used for the residual part [S6]. The bonded and non-bonded parameters for Li^+ and the remaining part were obtained from Jensen et al. [S7]. and Gouveia et al. [S8], respectively. The partial charges of UiO-66 were fitted from DFT calculations result. A cutoff of 12 Å was used for both van der Waals interactions and long-range correction (particle-particle-particle-mesh) of Coulombic interactions.

The initial configurations were first minimized by conjugated-gradient energy minimization scheme employing a convergence criterion of 1.0×10^{-4}. The systems were then equilibrated in NPT ensemble using the Parrinello-Rahman barostat for 2 ns to maintain a temperature of 1000 K and a pressure of 1 atm. Another 5 ns production run in NPT ensemble at 1000 K was conducted finally. A time step of 0.2 fs was used for all simulations. Only the final 5 ns was sampled for radical distribution function (RDF) calculation and mean square displacement (MSD) calculation.

S2 Supplementary Figures and Tables

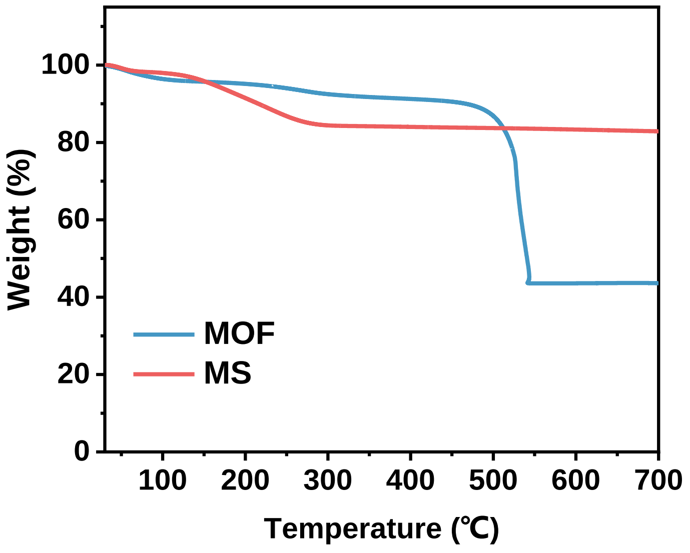

Fig. S1 TG profiles of MOF and MS particles

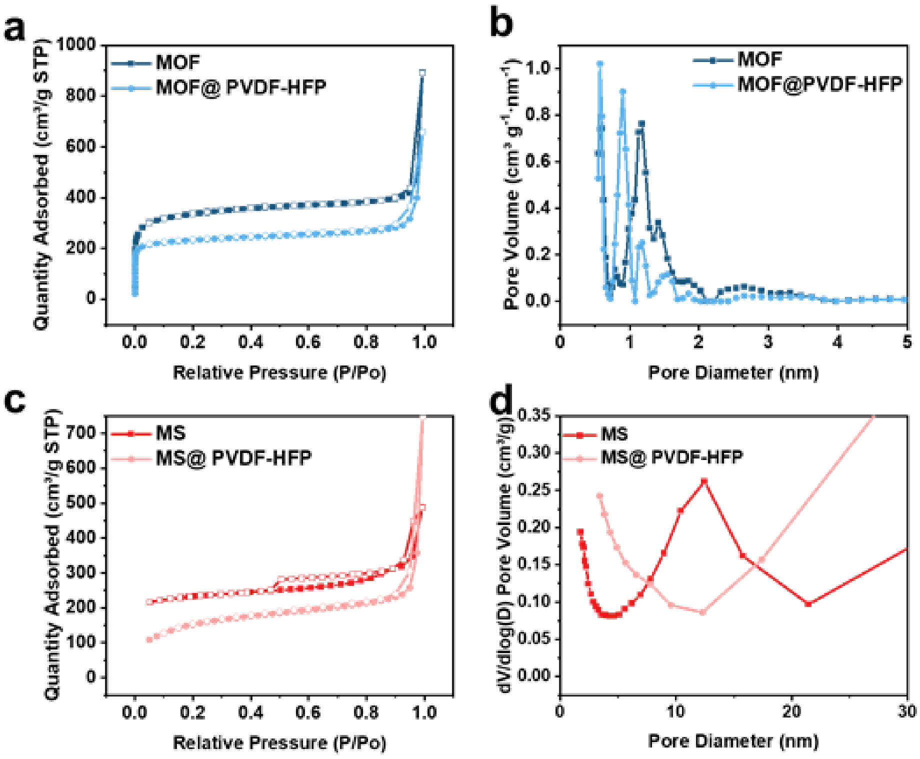

Fig. S2 N_2 adsorption-desorption isotherms and pore size distribution of a, b) MOF particles and c, d) MS particles before and after mixing with PVDF-HFP

Table S1 Pore size, surface area, and pore volume of MOF particles and MS particles before and after mixing with PVDF-HFP based on Nitrogen adsorption–desorption measurements

	**Pore size (nm)**	**Surface area (m^2/g)**	**Pore volume (cm^3/g)**
MOF	0.6 / 1.2	1130	0.62
MS	12	725	0.76
MOF@PVDF-HFP	0.6/0.9	878	0.518
MS@PVDF-HFP	/	374	0.431

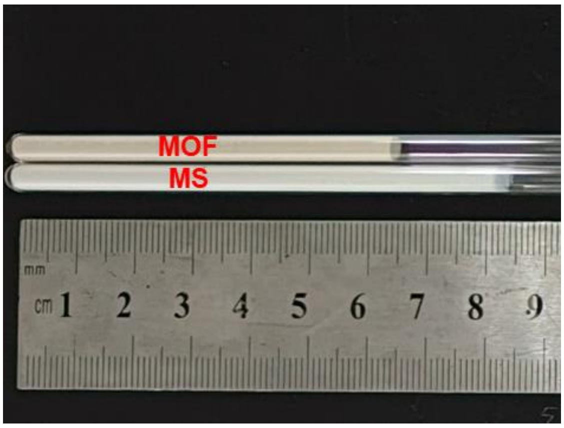

Fig. S3 Illustration of MOF and MS particles with different tap densities (500 mg)

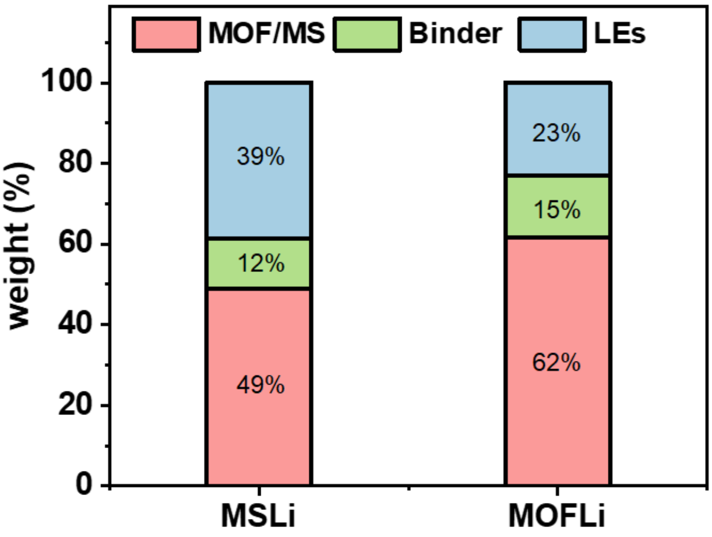

Fig. S4 The mass ratio of MOFLi QSEs and MSLi QSEs

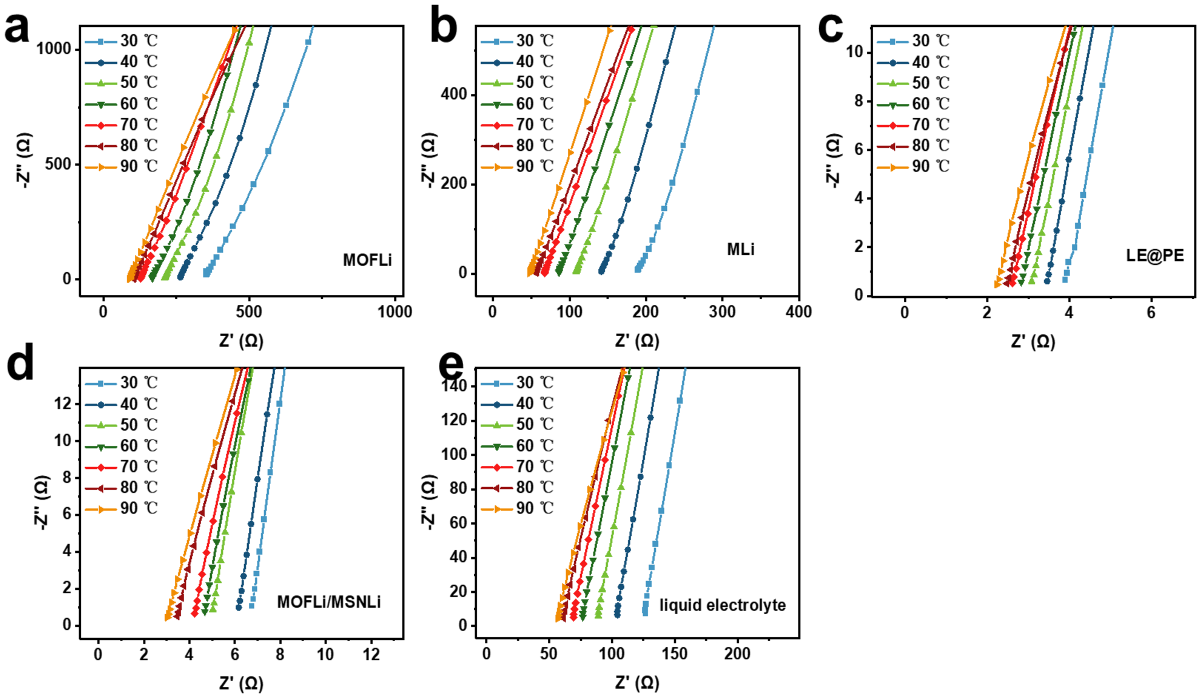

Fig. S5 Nyquist plots of stainless steel/electrolyte/ stainless steel cells with a) MOFLi QSEs (diameter: 10 mm, thickness: 504 μm), b) MSLi QSEs (diameter: 10 mm, thickness: 684 μm), c) liquid electrolyte in PE membrane (LE@PE) (diameter: 16 mm, thickness: 20 μm), and d) MOFLi/MSLi QSEs (diameter: 16 mm, thickness: 29 μm), e) pure liquid electrolyte (diameter: 10 mm, thickness 5500 μm) under different temperatures ranging from 30 to 90 °C

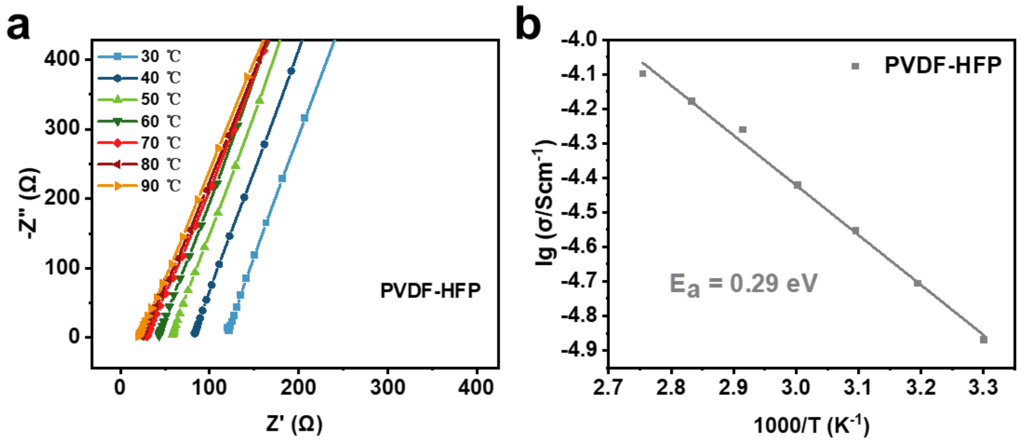

Fig. S6 a) Nyquist plots and b) corresponding Arrhenius plots of stainless steel|PVDF-HFP QSE|stainless steel cells. A free-standing PVDF-HFP membrane was prepared by casting and drying the solution of PVDF-HFP in DME. The PVDF-HFP membrane was then immersed in a 1 M LiTFSI in PC solution for 12 h to obtain the PVDF-HFP QSE (diameter: 16 mm, thickness: 32 μm)

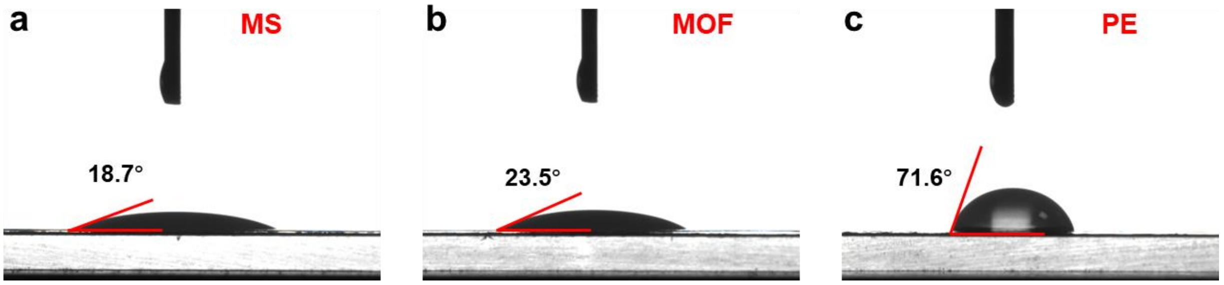

Fig. S7 Contact angle measurements of a) MS b) MOF and c) commercial PE with LEs (1 M LiTFSI in PC solution)

Table S2 Porosity measurement of PE and MOF or MS pellets

	**thickness (μm)**	**diameter (mm)**	**porous matrix volume (mm^3)**	**absorbed LEs (μL)**	**porosity (%)**
**PE**	20	18	5.09	1.79	35
**MOF pellet**	1477	10	116	32.4	28
**MS pellet**	1870	10	147	72.2	49

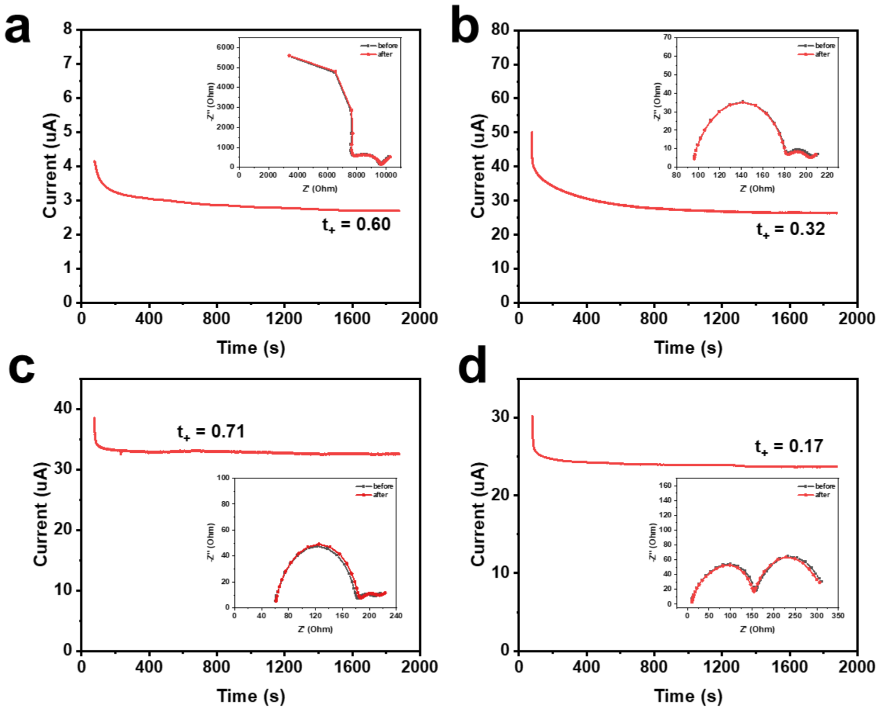

Fig. S8 The I-t curves and the corresponding EIS plots (inset) of Li||Li symmetric cells when equipped with a) MOFLi QSEs, b) MSLi QSEs, c) Janus MOFLi/MSLi QSEs, and d) liquid electrolytes in PE membrane

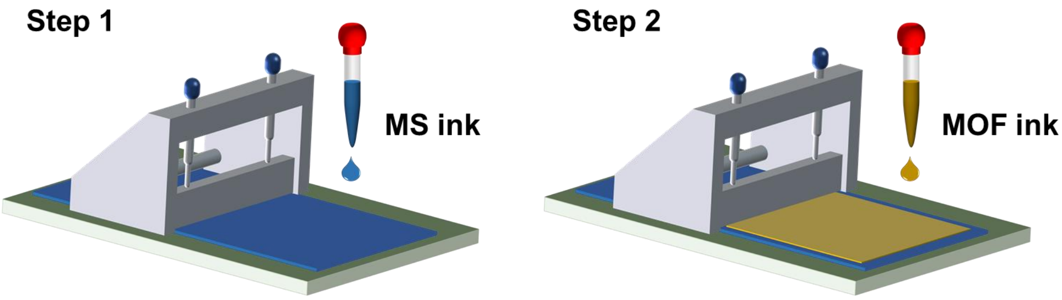

Fig. S9 Schematic illustration of the preparation of the Janus MOF/MS membrane

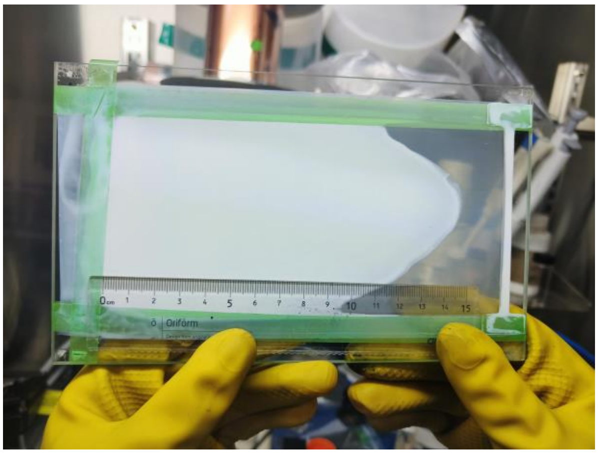

Fig. S10 Large-area fabrication of Janus MOF/MS membrane

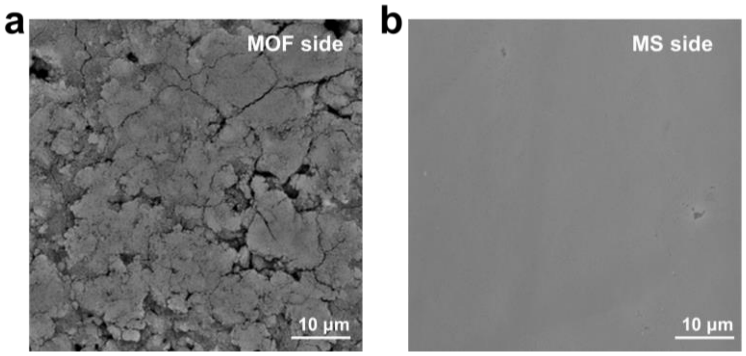

Fig. S11 SEM image of the a) MOF side and b) MS side of the MOF/MS membrane

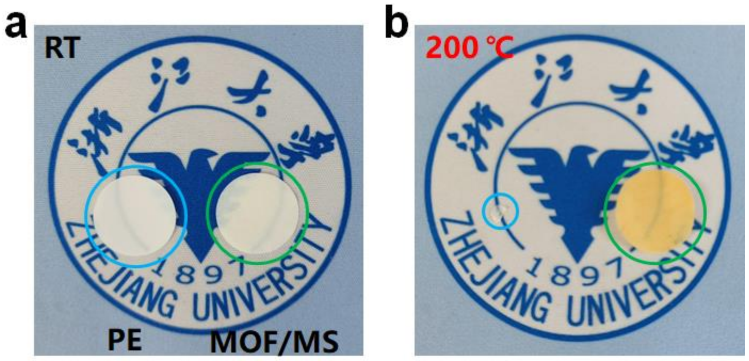

Fig. S12 High-temperature stability test of PE membrane and MOF/MS membrane

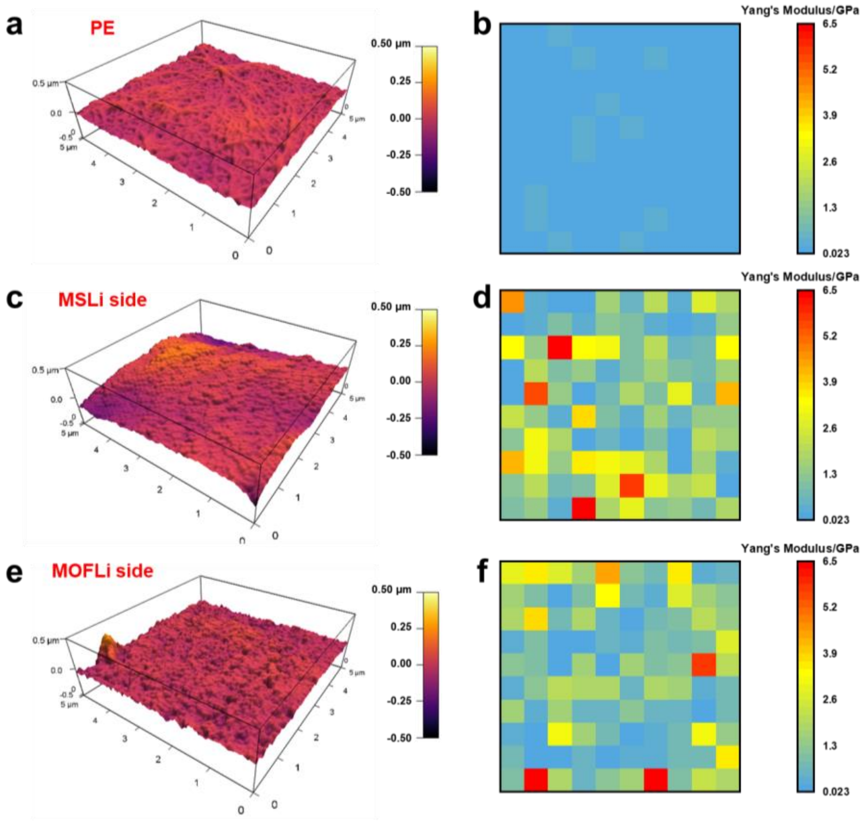

Fig. S13 3D surface morphology and Yang's modulus testing of (a-b) commercial PE membrane, (c-d) MSLi side and (e-f) MOFLi side of MOFLi/MSLi QSEs by AFM

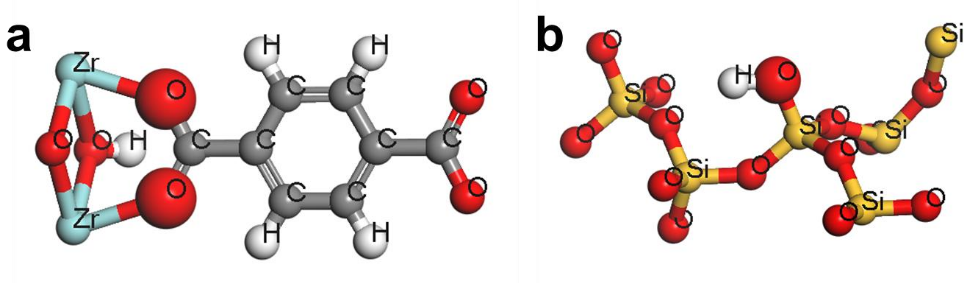

Fig. S14 Illustration of Lewis-base atom of a) O(Zr-O-C) and b) O(Si-O-Si)

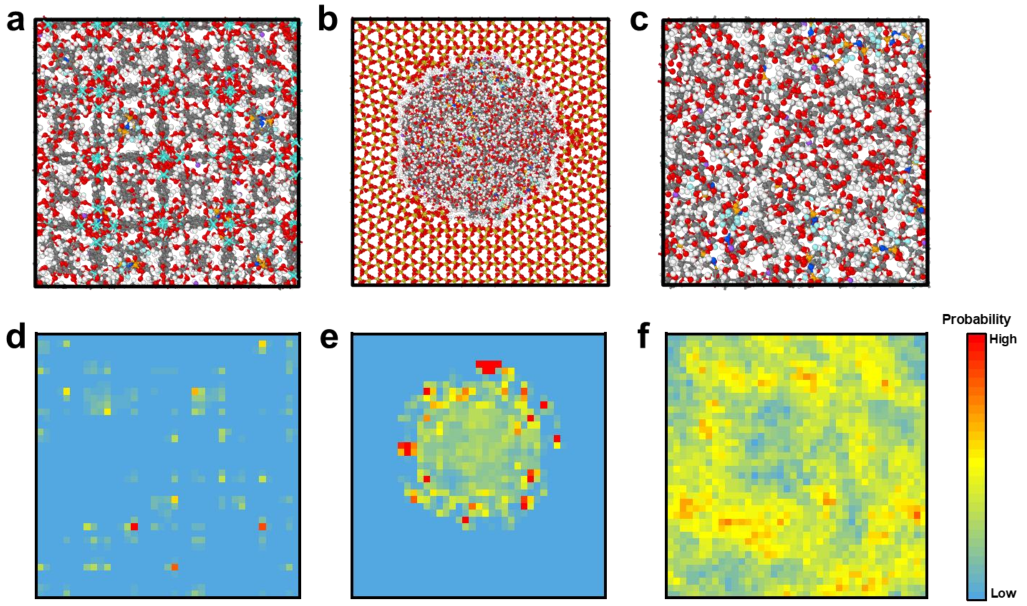

Fig. S15 (a-c) The final snap of the models and (d-f) the distribution of Li^+ during the whole simulation procedure of a, d) MOFLi, b, e) MSLi and c, f) LEs

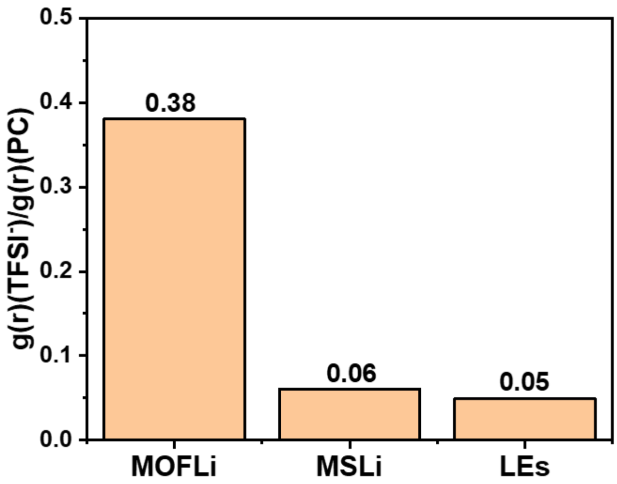

Fig. S16 The ration between the peak values of g(r) belonging to O($TFSI^-$) and O(PC)

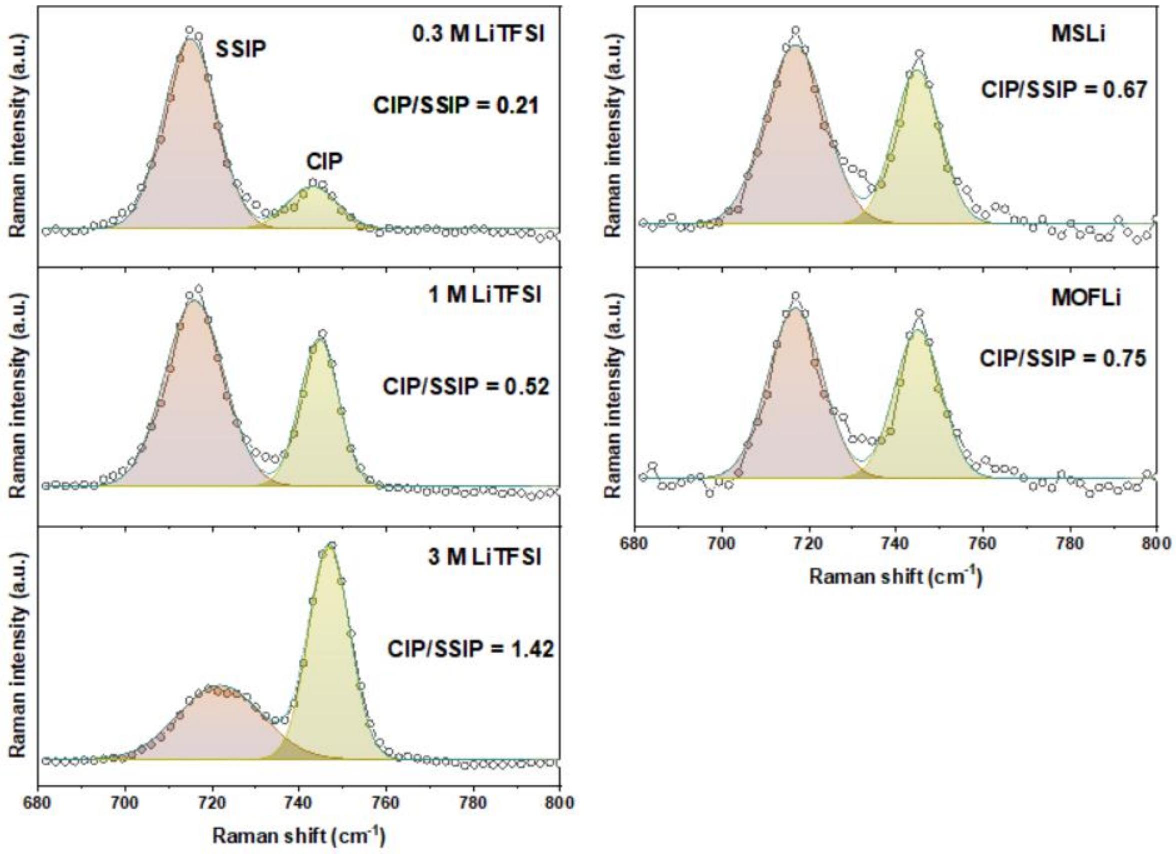

Fig. S17 Raman spectra of LEs with different concentration, MSLi QSEs, and MOFLi QSEs

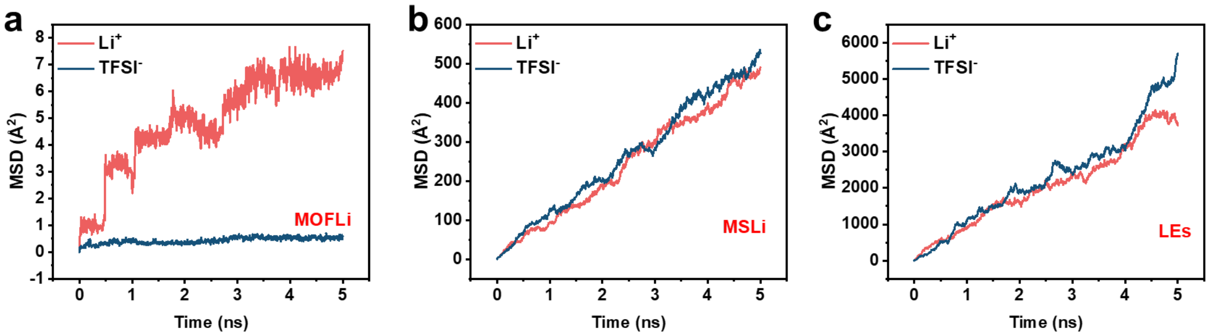

Fig. S18 Mean squared displacement (MSD) of a) MOFLi, b) MSLi and c) LEs

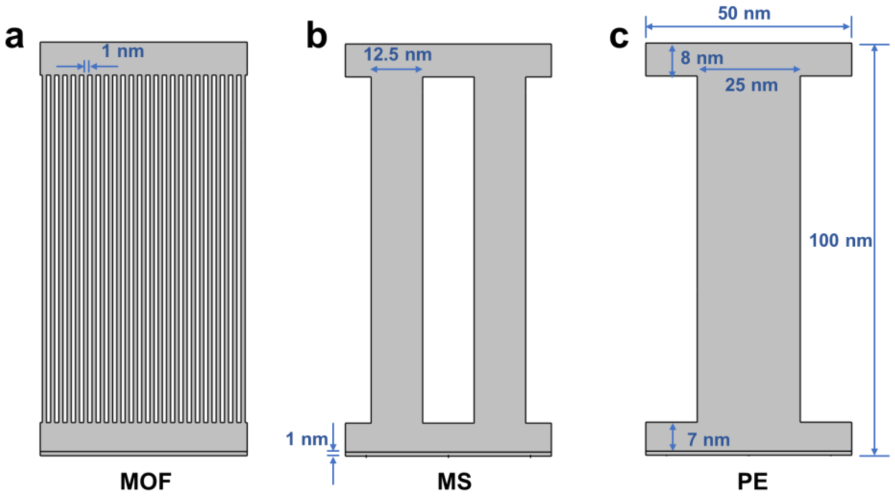

Fig. S19 Illustration and geometry parameters of the model equipped with a) MOFLi QSEs, b) MS QSEs, and c) PE membrane for COMSOL simulation

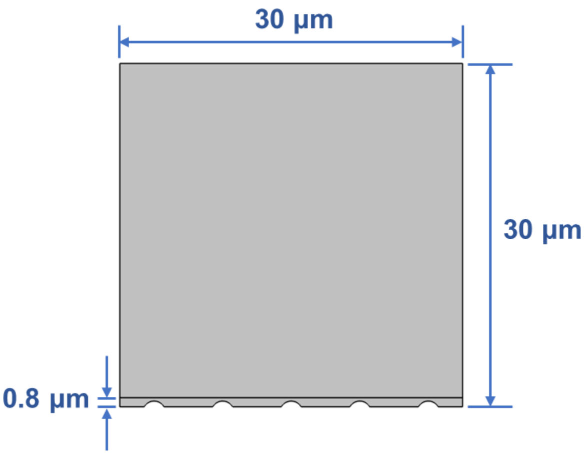

Fig. S20 Illustration and geometry parameters of the model for simulating Li-ion deposition

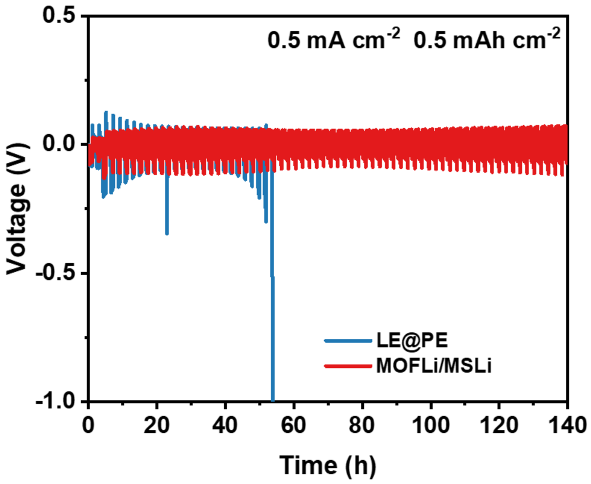

Fig. S21 Cycling performance of Li||Li cells with PE membrane and MOFLi/MSLi QSEs with 1 M LiTFSI/PC electrolyte

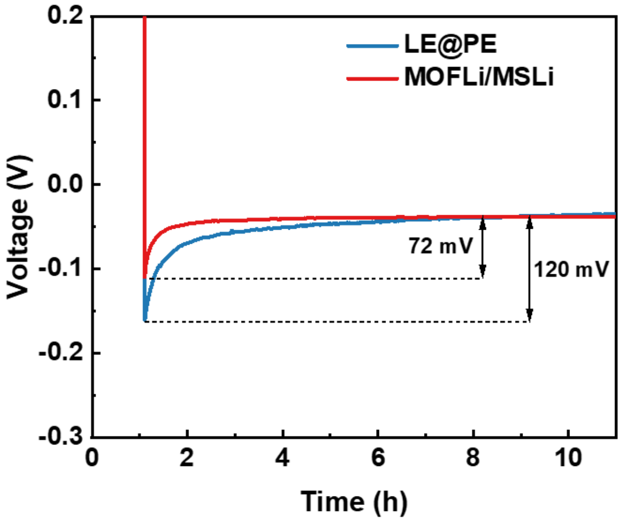

Fig. S22 Nucleation potential under a current density of 0.5 mA cm^{-2}

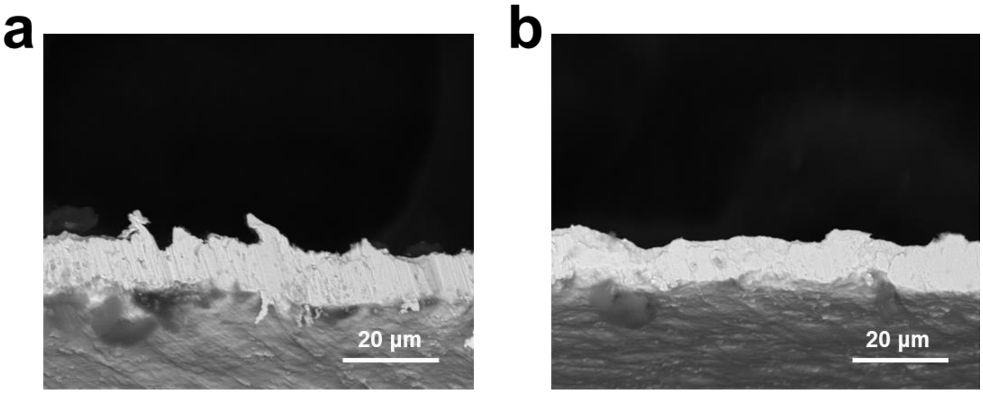

Fig. S23 The cross-section view of the deposited lithium on Cu when using a) PE membrane and b) MOFLi/MSLi QSEs

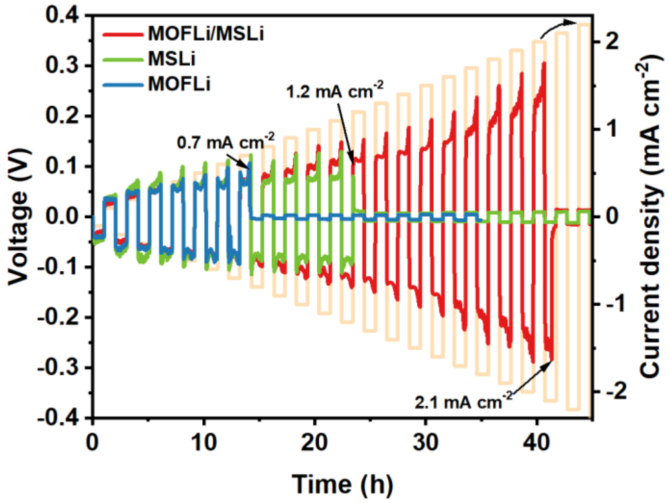

Fig. S24 Critical current density (CCD) of asymmetric cells with MOFLi QSEs, MS QSEs and MOFLi/MSLi QSEs

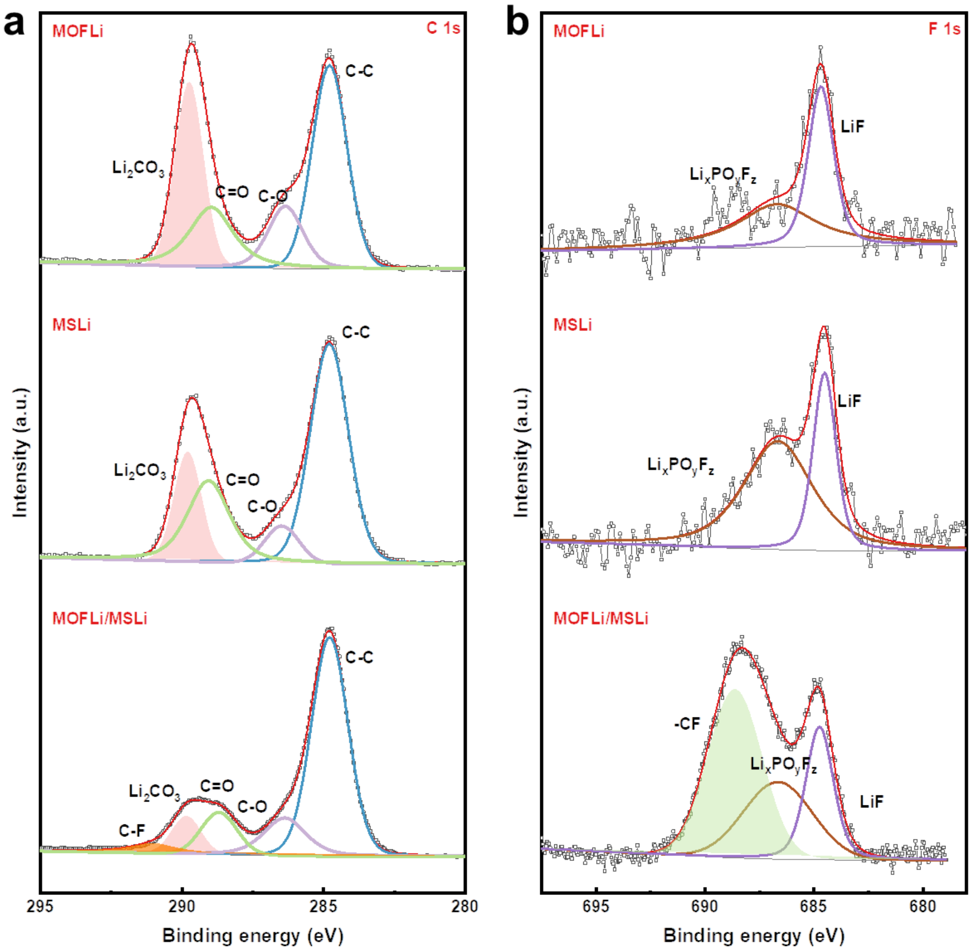

Fig. S25 X-ray photoelectron spectroscopy of lithium anode after cycling under 0.5 mA cm^{-2} with a capacity of 0.5 mA h cm^{-2} for 10 cycles: a) C 1s spectra; b) F 1s spectra

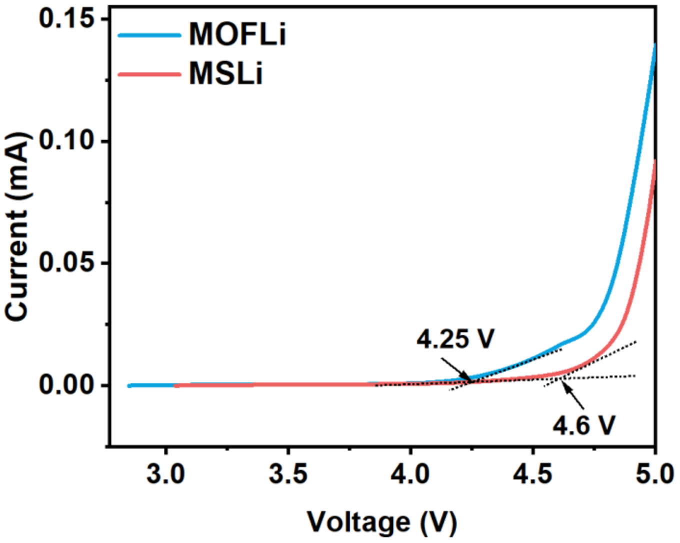

Fig. S26 LSV curves of MOFLi and MSLi QSEs

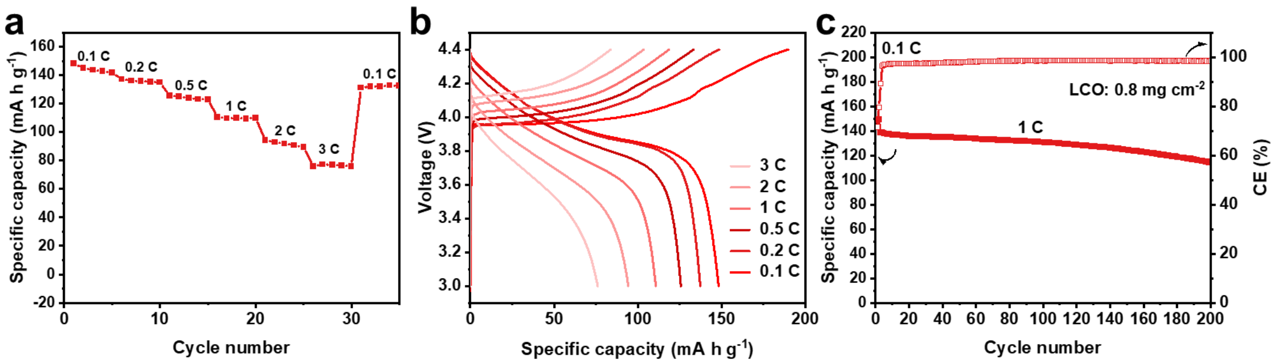

Fig. S27 Performance of LCO||Li batteries. **a, b**) Rate performance of LCO|MOFLi/MSLi|Li batteries and the corresponding voltage profile. **c**) Cycling performance of LCO||Li batteries at 1 C

Supplementary References

[S1]Y. Xu, L. Gao, L. Shen, Q. Liu, Y. Zhu et al., Ion-transport-rectifying layer enables li-metal batteries with high energy density. Matter **3**(5), 1685-1700 (2020). https://doi.org/10.1016/j.matt.2020.08.011

[S2]G. Wang, J. Gao, Y. Fu, Z. Ren, J. Huang et al., Implantable composite fibres with self-supplied h2o2 for localized chemodynamic therapy. Chem. Eng. J. **388**, 124211 (2020). https://doi.org/10.1016/j.cej.2020.124211

[S3]S. Plimpton, Fast parallel algorithms for short-range molecular dynamics. J. Comput. Phys. **117**, 1-19 (1995). https://doi.org/10.1006/jcph.1995.1039

[S4]W. L. Jorgensen, D. S. Maxwell, J. Tirado-Rives, Development and testing of the opls all-atom force field on conformational energetics and properties of organic liquids. **118**, 11225-11236 (1996). https://doi.org/10.1021/ja9621760

[S5]G. A. Kaminski, R. A. Friesner, J. Tirado-Rives, W. L. Jorgensen, Evaluation and reparametrization of the opls-aa force field for proteins via comparison with accurate quantum chemical calculations on peptides. J Phys Chem B. **105**, 6474-6487 (2001). https://doi.org/10.1021/jp003919d

[S6]A. K. Rappe, C. J. Casewit, K. S. Colwell, W. A. Goddard, W. M. Skiff, Uff, a full periodic table force field for molecular mechanics and molecular dynamics simulations. J Am Chem Soc. **114**, 10024-10035 (1992). https://doi.org/10.1021/ja00051a040

[S7]K. P. Jensen, W. L. Jorgensen, Halide, ammonium, and alkali metal ion parameters for modeling aqueous solutions. J Chem Theory Comput. **2**(6), 1499-1509 (2006). https://doi.org/10.1021/ct600252r

[S8]A. S. L. Gouveia, C. E. S. Bernardes, L. C. Tome, E. I. Lozinskaya, Y. S. Vygodskii et al., Ionic liquids with anions based on fluorosulfonyl derivatives: From asymmetrical substitutions to a consistent force field model. Phys Chem Chem Phys. **19**(43), 29617-29624 (2017). https://doi.org/10.1039/c7cp06081e
